# Pharmacokinetic analysis and steady-state predictions of different preparations of metronidazole administered per rectum in adult horses

**DOI:** 10.1093/jvimsj/aalaf032

**Published:** 2026-01-21

**Authors:** Jenni R E Auvinen, Janice E Kritchevsky, Jennifer M Reinhart, Alexandria E Gochenauer, Amber S Jannasch, Yu Han-Hallett

**Affiliations:** Department of Veterinary Clinical Sciences, College of Veterinary Medicine, Purdue University, West Lafayette, IN 47907, United States; Department of Veterinary Clinical Sciences, College of Veterinary Medicine, Purdue University, West Lafayette, IN 47907, United States; Department of Veterinary Clinical Medicine, University of Illinois Urbana-Champaign, Urbana, IL 61802, United States; Department of Veterinary Clinical Sciences, College of Veterinary Medicine, Purdue University, West Lafayette, IN 47907, United States; Bindley Bioscience Center, Purdue University, West Lafayette, IN 47907, United States; Bindley Bioscience Center, Purdue University, West Lafayette, IN 47907, United States

**Keywords:** absorption, bioavailability, equine, flagyl, rectal

## Abstract

**Background:**

Manipulation of forms of rectally administered metronidazole to improve bioavailability in horses has not been reported.

**Hypothesis/Objectives:**

Evaluate the pharmacokinetics of 3 rectal metronidazole preparations compared to nasogastric (NG) administration.

**Animals:**

Seven healthy horses.

**Methods:**

Phase 1A was a randomized, 3-way crossover, single-dose pharmacokinetic study, and Phases 1B and 2 were non-randomized, single-dose follow-up studies. Metronidazole (20 mg/kg) was administered NG and rectally in water (RW20), as a rectal gel (RG), and in dimethyl sulfoxide (DMSO). Metronidazole (80 mg/kg) was also administered rectally in water (RW80) to 3 horses. Plasma concentrations were measured using liquid chromatography/tandem mass spectrometry. Pharmacokinetic variables were calculated, and predicted steady-state area under the curve (AUC_0-24,ss_) to minimum inhibitory concentration ratio was used as a pharmacodynamic target.

**Results:**

Bioavailabilities for RW20 (33.7%), RG (2.49%), and DMSO (12.0%) were low relative to NG administration. When administered at a dosage of 20 mg/kg, only NG every 8 h was predicted to achieve the pharmacodynamic target in all horses. Administered rectally in water, the metronidazole maximum concentration increased from 3.11 +/− 0.63 μg/mL to 4.19 +/− 1.04 μg/mL when the dose was increased to 80 mg/kg. The RW80 predicted AUC_0-24,ss_ for every 8 h administration was above target for all 3 horses.

**Conclusions and clinical importance:**

With the tested preparations, rectal administration of metronidazole at a standard dosage of 20 mg/kg yielded subtherapeutic plasma concentrations. Administering a 4-fold higher dose rectally in water might overcome these limitations. Oral and intravenous routes remain the preferred methods for administering metronidazole in horses.

## Introduction

Metronidazole is a concentration-dependent, bactericidal nitroimidazole antibacterial and antiprotozoal agent commonly used in horses to treat anaerobic bacterial infections such as peritonitis, pleuropneumonia, and gastrointestinal disease.^1^^,^^2^ Oral administration of metronidazole in horses is a common practice and the pharmacokinetics are well understood.^3^^,^^4^ However, alternative drug delivery routes are required in horses with impaired gastrointestinal motility and ileus when oral administration is contraindicated. Intravenous metronidazole can be used to treat foals but is often cost-prohibitive in adult horses. Clinically, metronidazole tablets have been administered per rectum in adult horses as an alternative when oral administration is contraindicated or otherwise challenging because of horse compliance. In other veterinary species and in human medicine, bioavailability of rectal metronidazole has been reported to be similar to that of oral administration.^5–7^ However, in horses, rectal metronidazole administered as an aqueous solution at the dosage of 15-20 mg/kg body weight has poor bioavailability leading to subtherapeutic plasma concentrations.^4^^,^^8^^,^^9^ In a clinical setting, either increased dose or decreased dosing interval is frequently elected to counteract the documented poor absorption, although there are no publications demonstrating safety or efficacy.

In human medicine, different drug delivery systems, such as MucoLox ointment or VersaBase Gel, have been used to increase drug contact time on different mucosal surfaces, resulting in improved topical, rectal, and vaginal absorption.^10–12^ Alternative drug delivery systems or the use of different solvents have not been investigated in equine rectal drug delivery. In addition, to date, no studies have been published describing manipulation of the rectally administered metronidazole in attempt to increase absorption and bioavailability.

The primary objective of the study was to evaluate the relative bioavailabilities and pharmacokinetics of 3 rectal metronidazole preparations (in water, as a rectal gel [RG], and in dimethyl sulfoxide [DMSO]) compared to intragastric administration in adult horses administered at 20 mg/kg bodyweight. We hypothesized that the RG and DMSO would improve rectal absorption of metronidazole compared to administration as an aqueous solution. A secondary objective of the study was to evaluate the pharmacokinetics of rectal metronidazole in water administered at a 4-fold higher dosage (80 mg/kg). We hypothesized that this protocol would yield predicted therapeutic plasma concentrations at steady state.

## Materials and methods

### Study design and animals

Seven horses, aged 14 to 27 years old, were used in the study and consisted of 5 mares and 2 geldings. Breeds were 3 Tennessee Walking Horses, 1 Arabian, 1 Quarter Horse, and 2 Quarter Horse crosses. The study sample was selected from the Purdue University College of Veterinary Medicine teaching herd. The horses were determined to be healthy based on history and physical examination findings; horses with equine asthma controlled with environmental factors (no medications) were not excluded. None had a recent history of gastrointestinal disease. During the study, horses were housed in individual stalls from the night before and for 48 h after the administration of metronidazole. The horses were monitored at 2-6-h intervals for any adverse effects, including diarrhea or colic signs. Fecal production and consistency were monitored throughout the study period. In addition, physical examinations were performed at 12-h intervals.

The study was conducted in 2 phases. The first phase was divided into Phases 1A and 1B. Phase 1A was designed as a randomized, 3-way crossover pharmacokinetic study where 6 horses received 3 different metronidazole preparations either intragastrically (in water) or rectally (in water or in RG) at the same dosage (20 mg/kg) with a 1- to 3-week washout period in between the studies. Within 6 months in Phase 1B, a fourth metronidazole preparation with DMSO was administered rectally to 6 horses, 5 of which were used in Phase 1A.

The non-randomized Phase 2 was conducted 1 year after Phase 1B and consisted of a single treatment of a 4-fold increase in metronidazole dosage compared to the standard dosage of 20 mg/kg body weight, administered rectally mixed with tap water. The dosage selection was based on presumed linear metronidazole absorption kinetics and predicted therapeutic plasma concentrations from Phase 1. This phase was conducted using 3 horses also used in Phase 1A.

### Phase 1A and 1B: metronidazole preparation and administration

During Phase 1, the following drug preparations and delivery routes were used: (1) metronidazole 500 mg tablets (Viona Pharmaceuticals) mixed with 45 mL of warm tap water to make an aqueous suspension and administered nasogastrically (NG group) and rectally (RW20 group); (2) compounded metronidazole rectal gel (RG group); and (3) metronidazole 500 mg tablets first suspended in 45 mL of warm tap water and then mixed with 45 mL of 99% DMSO (FWI) and administered rectally (DMSO group).

The metronidazole RG was compounded by a licensed pharmacist (AG) at the Purdue University Veterinary Hospital pharmacy. To make 120 g of total product, 12 grams of metronidazole USP powder was weighed out and mixed with 10.8 g of VersaBase Gel (PCCA) and 97.2 g of MucoLox (PCCA) base. The prepared gel was run through an EXAKT 50 ointment mill 2 separate times until the powder was fully incorporated. After the milling process, the preparation was loaded into a syringe and weighed to determine the final concentration, 800 mg/mL. The final product was stored at controlled room temperature (20-25 °C) and was considered physically stable for a period of 30 days in accordance with USP < 795> guidelines for non-sterile compounding.^11^ All gel preparations were used within the 30-day period. The absence of visible phase separation or other macroscopic changes in color or consistency was noted.

Each metronidazole preparation was administered at a 20 mg/kg body weight dosage. The intragastric doses were administered via NG intubation. The tube was flushed with 1 L of water to ensure no metronidazole remained in the tube. The tube was visually inspected after removal. The rectal preparations were deposited approximately 30 cm into the rectum via a red rubber catheter. The catheter was flushed with either 30 mL of tap water (water solutions) or air (gel) to ensure the entire dose was administered. The tubes were weighed before and after administration to ensure the entire amount was successfully delivered. No manual evacuation was performed prior to drug administration. Any feces produced for the first 2 h after rectal administration was monitored for obvious drug depositions.

The horses were maintained on typical diets throughout the study period; access to pasture was not provided. Morning feeding was withheld until approximately 2 h after drug administration.

### Phase 2 metronidazole preparation and administration

During Phase 2, 3 study subjects received a 4-fold dosage (80 mg/kg body weight) of rectal metronidazole in aqueous solution (RW80 group). The 500 mg metronidazole tablets were prepared similarly to the NG and RW20 preparations in Phase 1A using 135 mL of warm tap water. The product was administered rectally, followed by 30 mL of water to ensure the entire dose was successfully administered.

### Blood sampling

Indwelling catheters were placed in either the left or right jugular vein using standard aseptic technique. Patency was maintained by flushing the catheters with heparinized saline. Prior to sample collection, 12 mL of blood was aspirated from the catheter and discarded. A 6-ml sample of blood was then collected into 2 evacuated EDTA tubes. Baseline samples were collected immediately prior to drug administration (time 0), followed by sampling at 10, 20, 30, 45, 60, 80, and 100 min, and 2, 2.5, 3, 3.5, 4, 5, 6, 8, 10, 12, 24, and 48 h after drug administration. Plasma was harvested by centrifugation and stored at −80 °C until analysis.

### Metronidazole assay

The metronidazole concentrations were measured by using liquid chromatography/tandem mass spectrometry detection (LC/MS/MS). The plasma metronidazole extraction and analysis was performed at the Bindley Bioscience Center (Purdue University) using methods modified from Ilomuanya et al.^12^ The plasma samples were thawed at room temperature and 0.1 mL of each sample was transferred into a new tube for liquid extraction. To each, 80 μL of 1 M NH_4_OH was added, followed by 100 ng of d4-metronidazole (Toronto Research Chemical # M338882). The deuterated (d4) metronidazole was added as an internal standard for the analysis and used for quantitation of metronidazole. The samples were vortexed for 30 s. Then, liquid–liquid extraction was performed by adding 0.5 mL of methyl tert-butyl ether and vortexing for 10 minutes at room temperature. The samples were centrifuged at 10 000 *×g* for 5 min. The upper layer was transferred to a new tube and dried in a rotary SpeedVac device for 2-4 h, without heat. The extracted samples were stored at −80 °C until ready for analysis. For analysis by LC/MS/MS, each sample was reconstituted in 75 μL of 20% methanol with 5 mM ammonium formate.

An Agilent 1290 Infinity II LC system coupled to an Agilent 6470 series QQQ mass spectrometer (MS/MS) was used to analyze samples (Agilent Technologies). A Waters Atlantis T3 2.1 mm × 50 mm, 3 μm column was used for LC separation (Waters Corporation). The buffers were (A) water + 5 mM ammonium formate and (B) acetonitrile. The linear LC gradient was as follows: time 0 min, 0% B; time 2 min, 0% B; time 10 min, 100% B; time 12 min, 100% B; time 12.1 min, 0% B; time 15 min, 0% B. The flow rate was 0.3 mL/min. Multiple reaction monitoring was used for MS analysis. The collision energy was set at 15 V for quantitation with the following transitions: metronidazole 172.4 m/z ➔ 128.1 m/z, metronidazole-d4 176.4 m/z ➔ 128.1 m/z. Data were acquired in positive electrospray ionization (ESI) mode. The jet stream ESI interface had a gas temperature of 325 °C, gas flow rate of 7 L/min, nebulizer pressure of 45 psi, sheath gas temperature of 250 °C, sheath gas flow rate of 7 L/min, capillary voltage of 4000 V in positive mode, and a nozzle voltage of 1000 V. The ΔEMV voltage was 300 V. The linear range of the assay was 1000 to 0.1 ng/mL. Agilent MassHunter Quantitative Analysis software was used for data analysis (version 10.1). The results were finally reported in units of μg/mL to allow comparison with previous studies.^9^

### Pharmacokinetic and statistical analysis

Using a commercially available power calculator (https://statulator.com/SampleSize/ss2PM.html#), it was determined that a sample size of 6 horses in repeated measures was sufficient to have 90% power and a level of significance of 0.05 to detect the difference in AUC of 20 ± 10 μg*h/mL. In addition, 6 horses were also sufficient to demonstrate a significant difference in *C*_max_ of 13 ± 6 μg/mL with 90% power and 95% confidence.

Continuous data are presented as geometric mean ± SD. Non-compartmental pharmacokinetic analysis was performed using Phoenix WinNonlin (Certara L.P.) for all phases. For Phase 1A, bioavailability relative to the NG route was calculated for each animal for each rectal preparation: *F_NG_* = (AUC_0-∞, rectal_ * Dose_NG_)/(AUC_0-∞, NG_ * Dose_rectal_) × 100%, where AUC_0-∞_ is the area under the curve extrapolated to infinity. Differences in terminal half-life (*t*_1/2,z_), time to maximum concentration (*T*_max_), maximum concentration (*C*_max_), area under the curve extrapolated to infinity (AUC_0-∞_), and mean residence time (MRT) between the NG, RW20, and RG treatments were evaluated using commercially available statistical software (Prism 9, GraphPad Software Inc.). First, data were assessed for normality using the Kolmogorov–Smirnov test. Then, variables were compared between phases using either a repeated measures ANOVA with Tukey’s multiple comparisons test or a Friedman test with Dunn’s multiple comparisons test, depending on normality. We elected not to compare variables between the DMSO treatment (Phase 1B) and other treatments (Phase 1A) because of study sample differences and the extended interval between study phases. Significance was set at *P* < .05.

To predict The 24-h AUC at steady state (AUC_0-24,ss_), the non-parametric superimposition function in WinNonLin was used predict drug concentrations after 48 h of multi-dose administration using both 8- and 12-h dosing intervals. The 48-h timepoint was selected because this was beyond 5 times the half-life for all horses and all metronidazole preparations after a single dose. A steady-state AUC_0-τ,ss_ was calculated for each dosing interval investigated using linear interpolation of the predicted concentrations. Then, the AUC_0-24,ss_ was calculated by multiplying the AUC_0-τ,ss_ by the number of doses administered within a 24-h period (3 doses for q 8 h dosing and 2 doses for q 12 h dosing). These steady-state predictions were compared to a target AUC_0-24,ss_ of at least 140-280 μg*h/mL, based on typical minimum inhibitory concentrations (MIC) of 2-4 μg/mL and a target AUC_0-24_/MIC ratio > 70.^2^^,^^13–15^

For Phase 1B, pharmacokinetic analysis and steady-state predictions were performed as described for Phase 1A. Relative bioavailability was only calculated for horses that had participated in Phase 1A (*n* = 3). No statistical comparisons were made. For Phase 2, pharmacokinetic analysis and steady-state predictions were performed as described for Phase 1A. Relative bioavailability and statistical comparisons were not performed.

## Results

### Animals

A total of 6 randomly selected horses were used for the initial study (Phase 1A), which all 6 completed. Horse 3 was unavailable for the DMSO trial (Phase 1B) because of unrelated reasons and was replaced by another, randomly selected horse 7. No adverse effects or abnormalities in physical examinations were noted during Phase 1A. In Phase 1B, 2 of the 6 horses (horses 1 and 5) defecated immediately upon administration of the dose, which was suspected to be caused by rectal irritation from the DMSO. These horses were excluded from analysis.

Three horses were included in Phase 2, all of which had participated in Phase 1A and Phase 1B. Horse 6 defecated within 1 h of administration of medication with mild, visual drug residue on feces. Horses 2 and 4 showed mild lethargy, inappetence, and mildly elevated rectal temperatures 24 h after administration of metronidazole. Both horses responded to 1 dose of flunixin meglumine (1.1 mg/kg IV) and no other adverse effects were observed. All 3 horses were included in the final analysis. Individual horse signalment and study participation data are provided in Supplementary Material.

### Phase 1: pharmacokinetics of metronidazole after NG, rectal water, RG, and DMSO administration (20 mg/kg)

**Figure 1:**
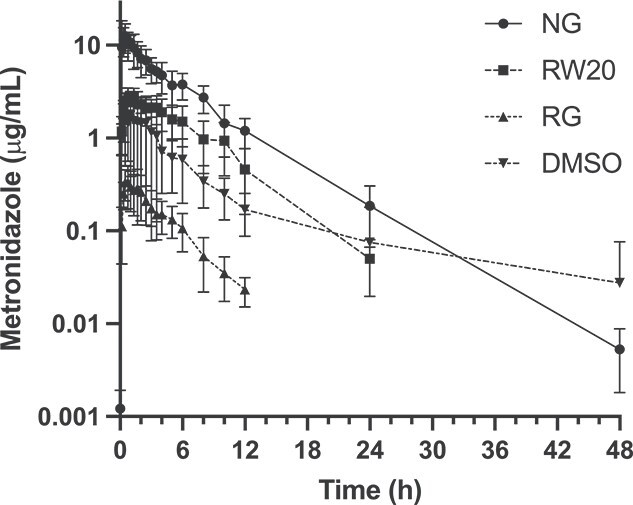
Plasma metronidazole time-concentrations profiles for NG, RW20, RG, and DMSO administration at 20 mg/kg (*n* = 6). Mean ± SD are shown. Note, metronidazole concentrations were undetectable for all horses in the RW20 group at 48 h and in the RG group at 24 and 48 h. Thus, these data cannot be presented on a semilogarithmic plot. Abbreviations: NG = nasogastric; RW20 = rectal water at 20 mg/kg; RG = rectal gel; DMSO = dimethyl sulfoxide.

The metronidazole plasma concentration profiles after NG, RW20, RG, and DMSO administration at a dosage of 20 mg/kg body weight are presented in Figure 1. The calculated pharmacokinetic variables for each treatment group are summarized in Table 1. Importantly, the bioavailabilities of the rectal preparations were only 3%-38% relative to NG administration.

**Table 1:** Pharmacokinetic variables of metronidazole after NG, RW20, RG, and DMSO administration at 20 mg/kg in horses (*n* = 6). Data are presented as geometric mean ± SD.

**Variable**	**Unit**	**NG**	**RW20**	**RG**	**DMSO**
λ_z_	1/h	0.155 +/− 0.014	0.155 +/− 0.012	0.153 +/− 0.044	0.139 +/- 0.068
*t*_1/2,z_	h	4.48 +/− 0.40	4.47 +/− 0.36	4.54 +/− 1.71	4.97 +/- 1.70
*T*_max_	h	0.389 +/− 0.335	1.142 +/− 1.302	0.612 +/− 0.180	0.577 +/− 0.385
*C*_max_	μg/mL	14.0 +/− 5.4	3.11 +/− 0.63	0.338 +/− 0.135	1.71 +/− 1.15
AUC_0-t_	μg*h/mL	60.7 +/− 17.1	20.4 +/− 7.97	1.51 +/− 0.68	9.84 +/− 5.44
AUC_0-∞_	μg*h/mL	60.7 +/− 17.1	20.4 +/− 7.97	1.51 +/− 0.68	10.1 +/− 5.5
AUC_%extrap_	%	0.046 +/− 0.036	0.063 +/− 0.020	0.368 +/− 0.191	0.604 +/− 3.284
AUMC_0-t_	μg*h^2^/mL	351 +/− 136	125 +/− 69	7.87 +/− 3.45	75.2 +/− 67.9
AUMC_0-∞_	μg*h^2^/mL	352 +/− 137	126 +/− 69	8.24 +/− 3.49	81.7 +/− 88.7
AUMC_%extrap_	%	0.428 +/− 0.247	0.559 +/− 0.217	3.71 +/− 2.17	4.13 +/− 8.36
MRT	h	5.80 +/− 0.99	6.17 +/− 0.86	5.44 +/− 0.55	8.13 +/− 7.01
*F*_NG_	%	–	33.7 +/− 17.9	2.49 +/− 1.31	12.0 +/− 7.96

Abbreviations: NG = nasogastric; RW20 = rectal water at 20 mg/kg; RG = rectal gel; DMSO = dimethyl sulfoxide; λ_z_ = terminal rate constant; *t*_1/2,z_ = terminal half-life; *T*_max_ = time at maximum concentration; *C*_max_ = maximum concentration; AUC_0-t_ = observed area under the curve; AUC_0-∞_ = area under the curve extrapolated to infinity; AUC_%extrap_ = % of the area under the curve extrapolated; AUMC_0-t_ = observed area under the moment curve; AUMC_0-∞_ = area under the moment curve extrapolated to infinity; AUMC_%extrap_ = % of the area under the moment curve extrapolated; MRT = mean residence time; *F*_NG_ = bioavailability relative to the nasogastric route.

**Figure 2:**
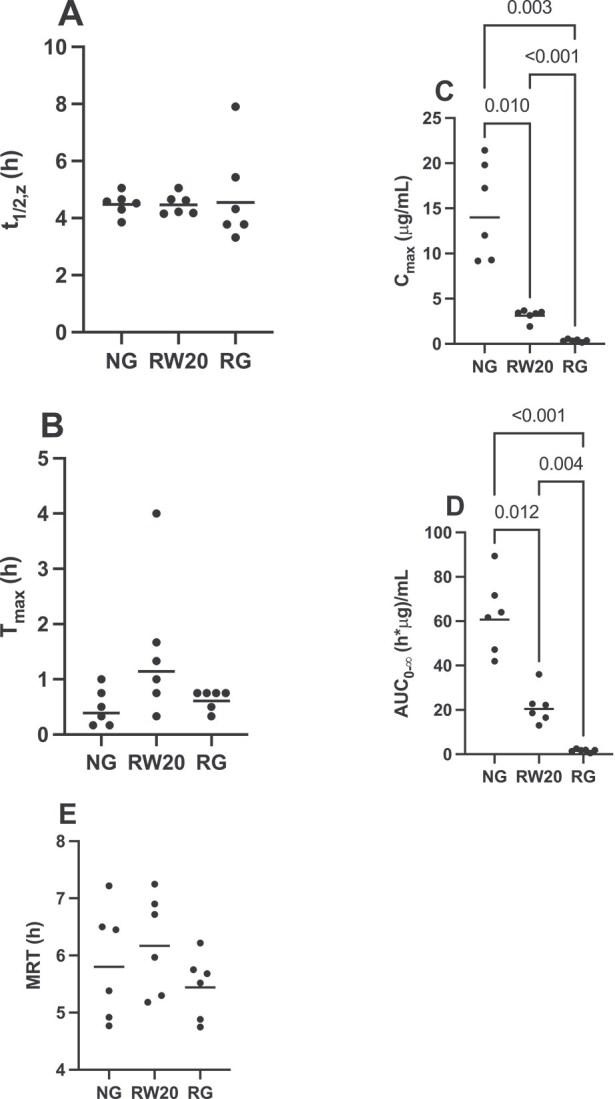
Comparison of *t*_1/2,z_ (A), *T*_max_ (B), *C*_max_ (C), AUC_0-∞_ (D), and MRT (E) after NG, RW, and RG administration (20 mg/kg) in horses (*n* = 6). Statistically significant pairwise comparisons are shown. The bar represents the geometric mean for each group. Abbreviations: NG = nasogastric; RW20 = rectal water at 20 mg/kg; RG = rectal gel; MRT = mean residence time.

Statistical analysis revealed significant differences in *C*_max_ (*P* = .002) and AUC (*P* < .001) with NG having the highest values, followed by RW20, followed by RG for both variables. *T*_max_ was also statistically different (*P* = .050) among the NG, RW20, and RG groups but post hoc testing revealed no significant pairwise comparisons. There were no significant differences in *t*_1/2,z_ (*P* = .759) or MRT (*P* = .217) among groups (Figure 2).

Steady-state predictions based on single-dose AUC_0-24,ss_ values are presented in Table S10. In the NG group, administration of metronidazole every 8 h resulted in predicted AUC_0-24,ss_ values ranging from 299 to 690 μg*h/mL, achieving the target (AUC_0-24,ss_ ≥ 140-280 μg*h/mL) in all horses. In contrast, administration every 12 h yielded predicted AUC_0-24,ss_ values ranging from 127 to 292 μg*h/mL, with only 4 of 6 horses reaching the minimum target in this group. In the RW20 group, 3 of 6 horses were predicted to achieve the target when administering metronidazole every 8 h (AUC_0-24,ss_ range 14.2-323 μg*h/mL). For all other routes and dosing intervals, the predicted AUC_0-24,ss_ values were subtherapeutic in all horses. Individual horse pharmacokinetic variables and plasma concentration profiles are provided in Supplementary Material.

### Phase 2: pharmacokinetics of metronidazole after RW80 administration

**Figure 3:**
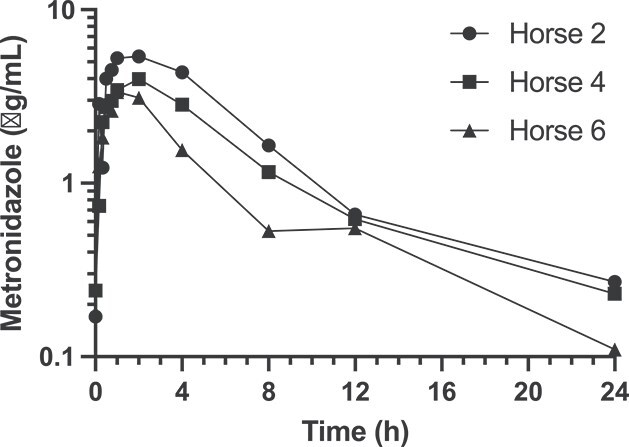
Plasma metronidazole concentration-time profiles after RW80 administration at 80 mg/kg in 3 horses. Abbreviation: RW80 = rectal water at 80 mg/kg.

The metronidazole plasma concentration profiles for individual horses (*n* = 3) after rectal administration in water at a dosage of 80 mg/kg body weight are presented in Figure 3. Pharmacokinetic variables and steady-state predictions for each horse are summarized in Table S11. Key values included *t*_1/2,z_ 5.96 +/− 1.05 h, *T*_max_ 1.59 +/− 0.58 h, *C*_max_ 4.16 +/− 1.04 μg/mL, AUC_0-t_ 29.0 +/− 10.2 μg*h/mL, and AUC_0-∞_ 30.7 +/− 10.7 μg*h/mL. Predicted AUC_0-24,ss_ were above the minimum target of 140 μg*h/mL for metronidazole administered every 8 h but at or below the target when administered every 12 h.

## Discussion

This study evaluated the single-dose pharmacokinetics of metronidazole after rectal administration of different preparations in horses with a goal of improving bioavailability. The study demonstrates that per rectum administration, regardless of formulation as an RG in DMSO or at an increased dose in water, results in poor and unpredictable bioavailability compared to NG administration. None of the rectally administered preparations achieved therapeutic predicted steady-state concentrations to treat common anaerobic infections when administered at the standard dosage of 20 mg/kg. However, a 4-fold increase in the metronidazole dosage (80 mg/kg) administered in rectal water might be sufficient to achieve therapeutic concentrations when administered every 8 h.

An AUC_0-24_:MIC ratio > 70 has been recommended as a pharmacodynamic target for metronidazole to allow adequate antimicrobial effect.^15^ No concrete data has been published for the common equine anaerobic bacteria, but MICs between 2 and 4 μg/mL have been suggested.^13^^,^^14^ Therefore, we aimed to achieve a predicted plasma metronidazole AUC_0-24,ss_ of at least 140-280 μg*h/mL at steady state. Metronidazole is poorly protein bound (<4%); therefore, protein binding was considered to have negligible importance in calculating tissue concentrations or pharmacologic effects.^16^

All horses in our study were predicted to have an AUC_0-24,ss_ above the target after NG administration of a single dose of metronidazole at 20 mg/kg body weight every 8 h. However, when the dosing interval was increased to every 12 h, the predicted AUC_0-24,ss_ fell below the target for 2 horses. This suggests that dosing every 8 h might be more appropriate for oral administration of metronidazole in this species. Regardless of the preparation (RW20, RG, or DMSO), rectal administration of standard doses of metronidazole resulted in substantially lower plasma concentrations compared to NG administration. While rectal administration of metronidazole in water at 20 mg/kg (RW20) demonstrated slightly better absorption than the RG or DMSO preparations, the overall relative bioavailability was poor compared to NG delivery. Furthermore, none of the rectal formulations were predicted to achieve therapeutic AUC_0-24,ss_ at steady state. All horses included in the study were clinically healthy with no evidence of gastrointestinal disease. Whether inflammation associated with colitis or other gastrointestinal disease influences drug absorption is unknown, and further investigation is needed in diseased horses.

Although it is known that metronidazole administered rectally in water has poor bioavailability in horses, it was hoped that the RG preparation would improve absorption based on the hypothesis of increased mucosal contact time.^4^^,^^9^ Instead, the RG resulted in even lower *C*_max_ and AUC_0-∞_ compared to RW20. The seemingly counterintuitive finding warrants further investigation into the impact of specific preparation components on drug release and absorption. Similarly, the lack of enhanced bioavailability with the DMSO preparation and the poor tolerability of the carrier in some subjects highlight the complexity of optimizing rectal drug delivery in horses.

The lack of improvement in rectal bioavailability with the addition of DMSO as a potential absorption enhancer was unexpected. This suggests that the limited rectal absorption is likely not solely attributable to poor permeability across the rectal mucosa. Other factors, such as the inherent solubility of metronidazole, the duration of mucosal contact, or the possibility of saturable absorption mechanisms, might play a more crucial role in limiting drug uptake. The observed local irritation in some subjects after DMSO administration reinforces concerns regarding the practical application of DMSO in this context.

The need to determine safe and effective alternatives to metronidazole oral administration prompted us to expand the trial. As metronidazole in water (RW20) achieved the highest relative bioavailability (38%) of the rectal preparations, we elected to evaluate this preparation at a higher dosage in the hopes of achieving target plasma drug concentrations. Increasing the dosage from 20 mg/kg to 80 mg/kg yielded somewhat higher peak plasma concentrations (RW20 3.11 ± 0.63 μg/mL vs RW80 4.29 ± 1.04 μg/mL). However, plasma concentrations did not increase proportionally to dose, which could suggest a saturable absorption process for metronidazole within the equine rectum. This saturation could be related to limitations in specific transport mechanisms or physiological factors governing drug uptake. Interestingly, increasing the dose by 4-fold in rectal water did achieve predicted AUC_0-24,ss_ above the therapeutic target when administered every 8 h, so this could be a reasonable approach for rectal metronidazole administration in horses. However, the AUC_0-24,ss_ for the RW80 group were still lower than those in the NG group and only 3 horses were included. Therefore, further evaluation is needed before this method can be recommended for general use. Two of the 3 horses became inappetent and developed mild rectal temperature elevations within 24 h of drug administration. These signs might reflect local irritation, toxicity, or proctitis, potentially resulting in secondary effects, including inflammatory cytokine release or transient bacteremia. Further evaluation of potential adverse effects is warranted, especially considering the observed inter-individual variations in rectal absorption across all preparations. Because the horses were of broadly similar ages, age is unlikely to have been a major contributor influencing variability. Instead, factors such as innate differences in colonic structure and function, prior parasite-related damage, regional blood flow, or other unrecognized effects could have played a role. Some degree of unexplained individual variation is also common in pharmacokinetic studies, even when underlying causes cannot be fully explained.

One of the limitations of this study was the small sample size in the initial pharmacokinetic phases. Furthermore, the Phase 2 study investigating the 4-fold metronidazole dose involved an even smaller group, limiting the generalizability of those specific findings. Although the study was designed as a crossover study, the unavailability of the same horses in all of the added phases, as well as the time gaps (6-12 months) between Phases 1A and 1B and between Phases 1 and 2, limited our ability to make direct statistical comparisons. Further studies with larger sample sizes, different age ranges and breeds, and more detailed investigations into the mechanisms of poor rectal absorption are warranted to confirm these observations and explore potential strategies to improve rectal bioavailability.

In conclusion, this study demonstrates poor and unreliable bioavailability of metronidazole after rectal administration in horses compared to the effective absorption via the gastric route. These findings have important clinical implications, suggesting that rectal administration of metronidazole, with the tested preparations, is not a reliable method for achieving therapeutic systemic drug concentrations in horses. Administering a 4-fold higher dose in rectal water might be a method to overcome these limitations but cannot be recommended without further study. Therefore, oral and intravenous administration remain the preferred routes for achieving therapeutic plasma concentrations of metronidazole in horses.

## Supplementary Material

aalaf032_Metronidazole_edited_Supplementary_Materials_10-22-25

## Off-label antimicrobial declaration

This study describes the use of metronidazole in horses; metronidazole is not currently approved for use in this species in the United States.

## Institutional animal care and use committee or other approval declaration

Approved by the Purdue University Institutional Animal Care and Use Committee (# 2002002009).

## Abbreviations

AUC: area under the curve
AUC_0-24,ss_: 24-hour AUC at steady state
*C*_max_: maximum concentration
DMSO: dimethyl sulfoxide
MIC: minimum inhibitory concentration
MRT: mean residence time
NG: nasogastric
RG: rectal gel
RW20: rectal water at 20 mg/kg
RW80: rectal water at 80 mg/kg
*T*_max_: time to maximum concentration
