# Supplementary material for: Pharmacokinetic analysis and steady-state predictions of different preparations of metronidazole administered per rectum in adult horses
Source: J Vet Intern Med. 2026 Jan 21;40(1):aalaf032. doi: 10.1093/jvimsj/aalaf032 (PMC12881942; doi:10.1093/jvimsj/aalaf032)
Supplement: aalaf032_Metronidazole_edited_Supplementary_Materials_10-22-25 [file aalaf032_metronidazole_edited_supplementary_materials_10-22-25.docx]

**Supplementary Materials**

**Table** **S1** – Plasma metronidazole concentrations (μg/mL) in horses administered a single dose 20 mg/kg metronidazole orally via nasogastric tube (NG).

| **Time (h)** | **Horse** | | | | | |
| --- | --- | --- | --- | --- | --- | --- |
|  | **1** | **2** | **3** | **4** | **5** | **6** |
| 0 | 0.00 | 0.00 | 0.00 | 0.00 | 0.00 | 0.00 |
| 0.17 | 2.91 | 8.01 | 21.42 | 19.80 | 2.85 | 2.12 |
| 0.33 | 9.18 | 16.06 | 15.86 | 12.66 | 7.14 | 7.95 |
| 0.5 | 8.20 | 17.23 | 16.82 | 15.37 | 7.49 | 10.48 |
| 0.75 | 6.88 | 14.71 | 13.15 | 6.26 | 8.68 | 12.01 |
| 1 | 7.79 | 13.22 | 12.62 | 10.21 | 9.28 | 11.27 |
| 1.33 | 5.15 | 14.53 | 10.80 | 4.98 | 8.66 | 11.91 |
| 1.67 | 6.72 | 9.87 | 11.01 | 4.17 | 8.02 | 10.07 |
| 2 | 5.04 | 8.87 | 10.22 | 2.98 | 7.99 | 7.73 |
| 2.5 | 5.59 | 8.47 | 8.44 | 3.24 | 7.06 | 8.33 |
| 3 | 4.91 | 7.49 | 6.68 | 2.54 | 5.58 | 6.32 |
| 3.5 | 4.88 | 7.07 | 6.36 | 3.70 | 5.15 | 4.18 |
| 4 | 3.10 | 7.21 | 6.49 | 2.90 | 4.24 | 4.54 |
| 5 | 1.65 | 5.92 | 5.03 | 2.67 | 3.32 | 3.62 |
| 6 | 2.96 | 4.98 | 3.90 | 5.38 | 2.51 | 2.88 |
| 8 | 1.87 | 3.83 | 2.55 | 3.64 | 1.61 | 2.92 |
| 10 | 0.77 | 2.63 | 0.57 | 2.21 | 1.04 | 1.43 |
| 12 | 0.78 | 1.85 | 1.17 | 1.41 | 0.69 | 1.28 |
| 24 | 0.07 | 0.33 | 0.13 | 0.27 | 0.05 | 0.27 |
| 48 | 0.00 | 0.01 | 0.00 | 0.01 | 0.00 | 0.00 |

**Table** **S2** – Plasma metronidazole concentrations (μg/mL) in horses administered a single dose 20 mg/kg metronidazole rectally dissolved in water (RW20).

| **Time (h)** | **Horse** | | | | | |
| --- | --- | --- | --- | --- | --- | --- |
|  | **1** | **2** | **3** | **4** | **5** | **6** |
| 0 | 0.00 | 0.00 | 0.00 | 0.00 | 0.00 | 0.00 |
| 0.17 | 1.29 | 0.82 | 1.85 | 1.73 | 0.58 | 0.79 |
| 0.33 | 2.61 | 1.85 | 1.93 | 2.53 | 2.19 | 1.38 |
| 0.5 | 3.22 | 2.06 | 1.90 | 3.10 | 2.55 | 2.27 |
| 0.75 | 2.84 | 1.79 | 1.85 | 3.14 | 3.35 | 3.42 |
| 1 | 3.67 | 2.03 | 1.79 | 3.05 | 2.03 | 2.10 |
| 1.33 | 3.22 | 2.66 | 1.69 | 3.22 | 3.37 | 2.75 |
| 1.67 | 2.97 | 2.44 | 1.78 | 3.52 | 1.18 | 1.90 |
| 2 | 2.47 | 2.49 | 1.62 | 2.69 | 2.68 | 1.86 |
| 2.5 | 2.34 | 2.04 | 1.53 | 3.44 | 1.48 | 1.70 |
| 3 | 2.02 | 2.15 | 1.57 | 2.36 | 2.88 | 1.83 |
| 3.5 | 1.67 | 1.85 | 1.45 | 3.46 | 1.92 | 2.38 |
| 4 | 1.72 | 3.15 | 1.25 | 2.31 | 1.62 | 1.34 |
| 5 | 1.32 | 0.95 | 1.12 | 2.78 | 1.79 | 1.60 |
| 6 | 1.27 | 0.99 | 0.94 | 2.85 | 1.49 | 1.49 |
| 8 | 0.84 | 0.61 | 0.55 | 2.07 | 0.87 | 0.89 |
| 10 | 1.45 | 0.44 | 0.33 | 1.54 | 1.26 | 0.58 |
| 12 | 0.43 | 0.23 | 0.20 | 1.05 | 0.44 | 0.40 |
| 24 | 0.07 | 0.02 | 0.02 | 0.09 | 0.07 | 0.03 |
| 48 | 0.00 | 0.00 | 0.00 | 0.00 | 0.00 | 0.00 |

**Table** **S3** – Plasma metronidazole concentrations (μg/mL) in horses administered a single dose 20 mg/kg metronidazole rectally in a gel formulation (RG).

| **Time (h)** | **Horse** | | | | | |
| --- | --- | --- | --- | --- | --- | --- |
|  | **1** | **2** | **3** | **4** | **5** | **6** |
| 0 | 0.00 | 0.00 | 0.00 | 0.00 | 0.00 | 0.00 |
| 0.17 | 0.14 | 0.07 | 0.14 | 0.06 | 0.22 | 0.04 |
| 0.33 | 0.33 | 0.24 | 0.29 | 0.24 | 0.31 | 0.10 |
| 0.5 | 0.33 | 0.43 | 0.51 | 0.30 | 0.27 | 0.16 |
| 0.75 | 0.37 | 0.44 | 0.56 | 0.33 | 0.20 | 0.11 |
| 1 | 0.33 | 0.38 | 0.50 | 0.26 | 0.24 | 0.09 |
| 1.33 | 0.32 | 0.28 | 0.46 | 0.31 | 0.21 | 0.07 |
| 1.67 | 0.35 | 0.37 | 0.55 | 0.23 | 0.17 | 0.06 |
| 2 | 0.23 | 0.41 | 0.44 | 0.22 | 0.15 | 0.12 |
| 2.5 | 0.23 | 0.29 | 0.34 | 0.18 | 0.13 | 0.09 |
| 3 | 0.19 | 0.22 | 0.31 | 0.20 | 0.09 | 0.04 |
| 3.5 | 0.17 | 0.17 | 0.24 | 0.18 | 0.10 | 0.04 |
| 4 | 0.17 | 0.17 | 0.24 | 0.14 | 0.11 | 0.07 |
| 5 | 0.14 | 0.13 | 0.19 | 0.15 | 0.15 | 0.04 |
| 6 | 0.12 | 0.09 | 0.14 | 0.15 | 0.12 | 0.02 |
| 8 | 0.07 | 0.03 | 0.09 | 0.08 | 0.04 | 0.01 |
| 10 | 0.05 | 0.02 | 0.05 | 0.05 | 0.03 | 0.01 |
| 12 | 0.03 | 0.02 | 0.03 | 0.03 | 0.02 | 0.01 |
| 24 | 0.00 | 0.00 | 0.00 | 0.00 | 0.00 | 0.00 |
| 48 | 0.00 | 0.00 | 0.00 | 0.00 | 0.00 | 0.00 |

**Table** **S4** – Plasma metronidazole concentrations (μg/mL) in horses administered a single dose 20 mg/kg metronidazole rectally in dimethyl sulfoxide (DMSO).

| **Time (h)** | **Horse** | | | |
| --- | --- | --- | --- | --- |
|  | **2** | **4** | **6** | **7** |
| 0 | 0.00 | 0.00 | 0.00 | 0.00 |
| 0.17 | 1.07 | 1.02 | 0.58 | 1.51 |
| 0.33 | 1.26 | 1.95 | 0.83 | 2.44 |
| 0.5 | 0.92 | 1.60 | 0.63 | 2.89 |
| 0.75 | 0.95 | 1.79 | 0.53 | 2.51 |
| 1 | 0.74 | 2.44 | 0.50 | 3.37 |
| 1.33 | 0.64 | 1.81 | 0.48 | 3.19 |
| 1.67 | 0.71 | 1.68 | 0.40 | 3.13 |
| 2 | 0.66 | 1.88 | 0.38 | 2.86 |
| 2.5 | 0.55 | 1.83 | 0.36 | 3.09 |
| 3 | 0.53 | 1.14 | 0.32 | 2.63 |
| 3.5 | 0.45 | 1.08 | 0.34 | 2.38 |
| 4 | 0.40 | 0.78 | 0.34 | 1.35 |
| 5 | 0.36 | 0.85 | 0.26 | 0.99 |
| 6 | 0.30 | 0.81 | 0.22 | 1.02 |
| 8 | 0.23 | 0.49 | 0.17 | 0.47 |
| 10 | 0.20 | 0.38 | 0.11 | 0.31 |
| 12 | 0.15 | 0.28 | 0.08 | 0.17 |
| 24 | 0.23 | 0.05 | 0.01 | 0.01 |
| 48 | 0.10 | 0.01 | 0.00 | 0.00 |

**Table** **S5** – Pharmacokinetic parameters and steady-state predictions for metronidazole in individual horses administered a single dose 20 mg/kg orally via nasogastric tube (NG).

| **Parameter** | **Unit** | **Horse** | | | | | |
| --- | --- | --- | --- | --- | --- | --- | --- |
|  |  | **1** | **2** | **3** | **4** | **5** | **6** |
| λ_z_ | 1/h | 0.161 | 0.149 | 0.151 | 0.137 | 0.180 | 0.153 |
| t_1/2,z_ | h | 4.3 | 4.6 | 4.6 | 5.1 | 3.9 | 4.5 |
| T_max_ | h | 0.3 | 0.5 | 0.2 | 0.2 | 1.0 | 0.8 |
| C_max_ | μg/mL | 9.2 | 17.2 | 21.4 | 19.8 | 9.3 | 12.0 |
| AUC_0-t_ | μg*h/mL | 42.0 | 89.3 | 71.6 | 61.6 | 47.2 | 64.1 |
| AUC_0-∞_ | μg*h/mL | 42.0 | 89.3 | 71.6 | 61.7 | 47.2 | 64.1 |
| AUC_%extrap_ | % | 0.0 | 0.1 | 0.0 | 0.1 | 0.0 | 0.0 |
| AUMC_0-t_ | μg*h^2^/mL | 225.1 | 577.9 | 350.0 | 441.5 | 224.7 | 411.9 |
| AUMC_0-∞_ | μg*h^2^/mL | 225.7 | 581.1 | 351.7 | 445.6 | 225.2 | 413.5 |
| AUMC_%extrap_ | % | 0.3 | 0.6 | 0.5 | 0.9 | 0.2 | 0.4 |
| MRT | h | 5.4 | 6.5 | 4.9 | 7.2 | 4.8 | 6.5 |
| F_NG_ | % | - | - | - | - | - | - |

λ_z_ = terminal rate constant; t_1/2,z_ = terminal half-life; T_max_ = time at maximum concentration; C_max_ = maximum concentration; AUC_0-t_ = observed area under the curve; AUC_0-∞_ = area under the curve extrapolated to infinity; AUC_%extrap_ = % of the area under the curve extrapolated; AUMC_0-t_ = observed area under the moment curve; AUMC_0-∞_ = area under the moment curve extrapolated to infinity; AUMC_%extrap_ = % of the area under the moment curve extrapolated; MRT = mean residence time; F_NG_ = bioavailability relative to the nasogastric route.

**Table** **S6** – Pharmacokinetic parameters and steady-state predictions for metronidazole in individual horses administered a single dose 20 mg/kg rectally dissolved in water (RW20).

| **Parameter** | **Unit** | **Horse** | | | | | |
| --- | --- | --- | --- | --- | --- | --- | --- |
|  |  | **1** | **2** | **3** | **4** | **5** | **6** |
| λ_z_ | 1/h | 0.137 | 0.149 | 0.164 | 0.166 | 0.150 | 0.167 |
| t_1/2,z_ | h | 5.0 | 4.6 | 4.2 | 4.2 | 4.6 | 4.2 |
| T_max_ | h | 1.0 | 4.0 | 0.3 | 1.7 | 1.3 | 0.8 |
| C_max_ | μg/mL | 3.7 | 3.2 | 1.9 | 3.5 | 3.4 | 3.4 |
| AUC_0-t_ | μg*h/mL | 22.6 | 16.5 | 13.1 | 36.0 | 22.2 | 18.6 |
| AUC_0-∞_ | μg*h/mL | 22.7 | 16.6 | 13.1 | 36.1 | 22.2 | 18.6 |
| AUC_%extrap_ | % | 0.1 | 0.1 | 0.1 | 0.0 | 0.1 | 0.0 |
| AUMC_0-t_ | μg*h^2^/mL | 150.9 | 85.1 | 68.8 | 260.4 | 152.3 | 110.9 |
| AUMC_0-∞_ | μg*h^2^/mL | 152.1 | 85.8 | 69.3 | 261.4 | 153.0 | 111.3 |
| AUMC_%extrap_ | % | 0.8 | 0.9 | 0.6 | 0.4 | 0.5 | 0.4 |
| MRT | h | 6.7 | 5.2 | 5.3 | 7.2 | 6.9 | 6.0 |
| F_NG_ | % | 54.0 | 18.5 | 18.2 | 58.4 | 47.0 | 29.1 |

λ_z_ = terminal rate constant; t_1/2,z_ = terminal half-life; T_max_ = time at maximum concentration; C_max_ = maximum concentration; AUC_0-t_ = observed area under the curve; AUC_0-∞_ = area under the curve extrapolated to infinity; AUC_%extrap_ = % of the area under the curve extrapolated; AUMC_0-t_ = observed area under the moment curve; AUMC_0-∞_ = area under the moment curve extrapolated to infinity; AUMC_%extrap_ = % of the area under the moment curve extrapolated; MRT = mean residence time; F_NG_ = bioavailability relative to the nasogastric route.

**Table** **S7** – Pharmacokinetic parameters and steady-state predictions for metronidazole in individual horses administered a single dose 20 mg/kg rectally in a gel formulation (RG).

| **Parameter** | **Unit** | **Horse** | | | | | |
| --- | --- | --- | --- | --- | --- | --- | --- |
|  |  | **1** | **2** | **3** | **4** | **5** | **6** |
| λ_z_ | 1/h | 0.128 | 0.088 | 0.209 | 0.184 | 0.183 | 0.161 |
| t_1/2,z_ | h | 5.4 | 7.9 | 3.3 | 3.8 | 3.8 | 4.3 |
| T_max_ | h | 0.8 | 0.8 | 0.8 | 0.8 | 0.3 | 0.5 |
| C_max_ | μg/mL | 0.4 | 0.4 | 0.6 | 0.3 | 0.3 | 0.2 |
| AUC_0-t_ | μg*h/mL | 1.9 | 1.8 | 2.6 | 1.9 | 1.4 | 0.5 |
| AUC_0-∞_ | μg*h/mL | 1.9 | 1.8 | 2.6 | 1.9 | 1.4 | 0.5 |
| AUC_%extrap_ | % | 0.4 | 0.6 | 0.1 | 0.4 | 0.3 | 0.7 |
| AUMC_0-t_ | μg*h^2^/mL | 10.7 | 8.1 | 12.0 | 11.3 | 7.5 | 2.7 |
| AUMC_0-∞_ | μg*h^2^/mL | 11.1 | 8.7 | 12.2 | 11.7 | 7.8 | 2.9 |
| AUMC_%extrap_ | % | 3.9 | 7.0 | 1.4 | 3.1 | 3.2 | 6.7 |
| MRT | h | 5.7 | 4.9 | 4.8 | 6.2 | 5.7 | 5.5 |
| F_NG_ | % | 4.6 | 2.0 | 3.6 | 3.1 | 2.9 | 0.8 |

λ_z_ = terminal rate constant; t_1/2,z_ = terminal half-life; T_max_ = time at maximum concentration; C_max_ = maximum concentration; AUC_0-t_ = observed area under the curve; AUC_0-∞_ = area under the curve extrapolated to infinity; AUC_%extrap_ = % of the area under the curve extrapolated; AUMC_0-t_ = observed area under the moment curve; AUMC_0-∞_ = area under the moment curve extrapolated to infinity; AUMC_%extrap_ = % of the area under the moment curve extrapolated; MRT = mean residence time; F_NG_ = bioavailability relative to the nasogastric route.

**Table** **S8** – Pharmacokinetic parameters and steady-state predictions for metronidazole in individual horses administered a single dose 20 mg/kg rectally in dimethyl sulfoxide (DMSO).

| **Parameter** | **Unit** | **Horse** | | | |
| --- | --- | --- | --- | --- | --- |
|  |  | **2** | **4** | **6** | **7** |
| λ_z_ | 1/h | 0.123 | 0.104 | 0.118 | 0.249 |
| t_1/2,z_ | h | 5.6 | 6.7 | 5.9 | 2.8 |
| T_max_ | h | 0.3 | 1.0 | 0.3 | 1.0 |
| C_max_ | μg/mL | 1.3 | 2.4 | 0.8 | 3.4 |
| AUC_0-t_ | μg*h/mL | 10.9 | 13.4 | 3.9 | 16.7 |
| AUC_0-∞_ | μg*h/mL | 11.7 | 13.5 | 3.9 | 16.7 |
| AUC_%extrap_ | % | 7.0 | 0.9 | 0.5 | 0.0 |
| AUMC_0-t_ | μg*h^2^/mL | 187.0 | 93.3 | 25.9 | 70.6 |
| AUMC_0-∞_ | μg*h^2^/mL | 232.8 | 100.0 | 27.0 | 71.0 |
| AUMC_%extrap_ | % | 19.7 | 6.7 | 4.0 | 0.6 |
| MRT | h | 19.9 | 7.4 | 7.0 | 4.3 |
| F_NG_ | % | 13.1 | 21.9 | 6.1 | - |

λ_z_ = terminal rate constant; t_1/2,z_ = terminal half-life; T_max_ = time at maximum concentration; C_max_ = maximum concentration; AUC_0-t_ = observed area under the curve; AUC_0-∞_ = area under the curve extrapolated to infinity; AUC_%extrap_ = % of the area under the curve extrapolated; AUMC_0-t_ = observed area under the moment curve; AUMC_0-∞_ = area under the moment curve extrapolated to infinity; AUMC_%extrap_ = % of the area under the moment curve extrapolated; MRT = mean residence time; F_NG_ = bioavailability relative to the nasogastric route.

**Table** **S9** – Plasma metronidazole concentrations (μg/mL) in horses administered a single dose 80 mg/kg metronidazole rectally dissolved in water (RW80).

| **Time (h)** | **Horse** | | |
| --- | --- | --- | --- |
|  | **2** | **4** | **6** |
| 0 | 0.17 | 0.24 | 0.18 |
| 0.17 | 2.87 | 0.74 | 1.25 |
| 0.33 | 1.23 | 2.25 | 1.83 |
| 0.5 | 4.00 | 2.81 | 2.75 |
| 0.75 | 4.49 | 2.98 | 2.62 |
| 1 | 5.25 | 3.45 | 3.36 |
| 2 | 5.39 | 3.99 | 3.11 |
| 4 | 4.35 | 2.83 | 1.55 |
| 8 | 1.65 | 1.16 | 0.53 |
| 12 | 0.66 | 0.62 | 0.55 |
| 24 | 0.27 | 0.23 | 0.11 |

**Table S10:** Predicted 24-hour area under the curves (AUC_0-24,ss_) of metronidazole when administered every 8 and 12 hours to healthy horses. The variables were compared to a target AUC_0-24,ss_ of at least 140-280 μg*h/mL based on a recommended AUC_0-24,ss_:MIC ratio > 70.

| **Horse** | **NG**  **(μg*h/mL)** | | **RW20**  **(μg*h/mL)** | | **RG**  **(μg*h/mL)** | | **DMSO**  **(μg*h/mL)** | |
| --- | --- | --- | --- | --- | --- | --- | --- | --- |
|  | *q 8 h* | *q 12 h* | *q 8 h* | *q 12 h* | *q 8 h* | *q 12 h* | *q 8 h* | *q 12 h* |
| 1 | 311 | 127 | 162 | 71.3 | 13.3 | 5.73 | - | - |
| 2 | 690 | 292 | 108 | 45.7 | 9.17 | 4.71 | 1.80 | 0.76 |
| 3 | 477 | 209 | 91.6 | 37.1 | 15.9 | 6.86 | - | - |
| 4 | 561 | 211 | 323 | 129 | 14.1 | 5.72 | 1.53 | 0.62 |
| 5 | 299 | 131 | 163 | 71.1 | 8.82 | 3.92 | - | - |
| 6 | 510 | 210 | 14.2 | 5.78 | 3.11 | 1.45 | 5.39 | 2.16 |
| 7 | - | - | - | - | - | - | 2.72 | 1.19 |

**Table S11:** Pharmacokinetic variables and steady-state predictions of metronidazole after RW80, administration at 80 mg/kg in horses (n=3). Data are presented as individual values and geometric mean ± standard deviation.

| **Variable** | **Units** | **Horse 2** | **Horse 4** | **Horse 6** | **Mean +/- SD** |
| --- | --- | --- | --- | --- | --- |
| λ_z_ | 1/h | 0.133 | 0.096 | 0.123 | 0.116 +/- 0.019 |
| t_1/2,z_ | h | 5.23 | 7.21 | 5.62 | 5.96 +/- 1.05 |
| T_max_ | h | 2.00 | 2.00 | 1.00 | 1.59 +/- 0.58 |
| C_max_ | μg/mL | 5.39 | 3.99 | 3.36 | 4.16 +/- 1.04 |
| AUC_0-t_ | μg*h/mL | 40.6 | 29.5 | 20.3 | 29.0 +/- 10.2 |
| AUC_0-∞_ | μg*h/mL | 42.6 | 31.9 | 21.2 | 30.7 +/- 10.7 |
| AUC_%extrap_ | % | 4.69 | 7.60 | 4.16 | 5.29 +/- 1.85 |
| AUMC_0-t_ | μg*h^2^/mL | 228 | 179 | 116 | 168 +/- 56 |
| AUMC_0-∞_ | μg*h^2^/mL | 291 | 263 | 144 | 223 +/- 78 |
| AUMC_%extrap_ | % | 21.7 | 31.8 | 19.6 | 23.8 +/- 6.5 |
| MRT | h | 6.83 | 8.24 | 6.81 | 7.26 +/- 0.82 |
| AUC_0-24,ss,q8_ | μg*h/mL | 323 | 261 | 156 | 236 +/- 85 |
| AUC_0-24,ss,q12_ | μg*h/mL | 140 | 121 | 84 | 112 +/- 28 |

λ_z_ = terminal rate constant; t_1/2,z_ = terminal half-life; T_max_ = time at maximum concentration; C_max_ = maximum concentration; AUC_0-t_ = observed area under the curve; AUC_0-∞_ = area under the curve extrapolated to infinity; AUC_%extrap_ = % of the area under the curve extrapolated; AUMC_0-t_ = observed area under the moment curve; AUMC_0-∞_ = area under the moment curve extrapolated to infinity; AUMC_%extrap_ = % of the area under the moment curve extrapolated; MRT = mean residence time; AUC_0-24,ss,q8_ = predicted 24-hour area under the curve at steady state when dosed every 8 hours; AUC_0-24,ss,q12_ = predicted 24-hour area under the curve at steady state when dosed every 12 hours.

**Table** **S12** – Individual horse signalment and study participation data.

| **Horse number** | **Age**  (years) | **Breed** | **Gender** | **Participation** | | |
| --- | --- | --- | --- | --- | --- | --- |
|  |  |  |  | **Phase 1A** | **Phase 1B** | **Phase 2** |
| 1 | 22 | QH x TWH | Gelding | Yes | Eliminated | No |
| 2 | 25 | Arab | Gelding | Yes | Yes | Yes |
| 3 | 27 | QH x SB | Mare | Yes | No | No |
| 4 | 23 | TWH | Mare | Yes | Yes | Yes |
| 5 | 17 | QH | Mare | Yes | Eliminated | No |
| 6 | 14 | TWH | Mare | Yes | Yes | Yes |
| 7 | 22 | TWH | Mare | No | Yes | No |

QH = Quarter Horse; TWH = Tennessee Walking Horse, SB = Standardbred

**Figure** **S1** – Individual horse time-concentration graphs for nasogastric (NG) administration of 20 mg/kg metronidazole.

**Figure** **S2** – Individual horse time-concentration graphs for rectal administration of 20 mg/kg metronidazole in water (RW20).

**Figure** **S3** – Individual horse time-concentration graphs for rectal administration of 20 mg/kg metronidazole in gel (RG).

**Figure** **S4** – Individual horse time-concentration graphs for rectal administration of 20 mg/kg metronidazole in DMSO (DMSO).
